# On the Chemistry of Respiration

**Published:** 1851-07

**Authors:** 


*On the Chemistry of Respiration.*—Dr. Horn has published the results of his experiments on the chemistry of the respiratory process. (*Neue Medicinisch-Chirurgische Zeitung.*) The paper is recapitulated in the following conclusions:—

1. The longer air is retained in the lungs, the larger the quantity of carbonic acid contained.

2. The products of expiration undergo a regular hourly variation.

3. The expiration of carbonic acid increases after the ingestion of food or drink.

4. Children and growing persons exhale more carbonic acid than old people; men more than women, and persons of the sanguine temperament more than those of the lymphatic habit.

5. The use of alcohol increases the exhalation of carbon.

6. Pain, sedentary occupations, menstruation, inflammation, fevers, are so many causes of the diminution of carbonic acid.

7. The exanthemata cause its increase.

The author offers likewise to demonstrate,—

*a.* That the act of respiration, tends to the maintainance of animal heat, as well as to the purification of the blood.

*b.* That the act of respiration is in an opposite ratio to the activity of the renal and hepatic secretions.

*c.* The oxygen is not the only element of the atmosphere which is taken into the blood, the nitrogen is also absorbed and rejected in variable proportions.

*d.* Irritability increases in proportion to the amount of oxygen absorbed.—*Prov. Med. and Surg. Jour.*

